# The vicious cycle of chronic endometriosis and depression—an immunological and physiological perspective

**DOI:** 10.3389/fmed.2024.1425691

**Published:** 2024-09-06

**Authors:** Subuhi Sherwani, Mohd Wajid Ali Khan, Saravanan Rajendrasozhan, Khalid Al-Motair, Qayyum Husain, Wahid Ali Khan

**Affiliations:** ^1^Department of Biology, College of Sciences, University of Hail, Hail, Saudi Arabia; ^2^Medical and Diagnostic Research Center, University of Hail, Hail, Saudi Arabia; ^3^Department of Chemistry, College of Sciences, University of Hail, Hail, Saudi Arabia; ^4^Department of Biochemistry, Faculty of Life Sciences, Aligarh Muslim University, Aligarh, India; ^5^Department of Clinical Biochemistry, College of Medicine, King Khalid University, Abha, Saudi Arabia

**Keywords:** estrogen, endometriosis, estrogen receptor, inflammation, depression, immune imbalance

## Abstract

Endometriosis is a chronic, estrogen-dependent, proinflammatory disease that can cause various dysfunctions. The main clinical manifestations of endometriosis include chronic pelvic pain and impaired fertility. The disease is characterized by a spectrum of dysfunctions spanning hormonal signaling, inflammation, immune dysregulation, angiogenesis, neurogenic inflammation, epigenetic alterations, and tissue remodeling. Dysregulated hormonal signaling, particularly involving estrogen and progesterone, drives abnormal growth and survival of endometrial-like tissue outside the uterus. Chronic inflammation, marked by immune cell infiltration and inflammatory mediator secretion, perpetuates tissue damage and pain. Altered immune function, impaired ectopic tissue clearance, and dysregulated cytokine production contribute to immune dysregulation. Enhanced angiogenesis promotes lesion growth and survival. Epigenetic modifications influence gene expression patterns, e.g., HSD11B1 gene, affecting disease pathogenesis. Endometriosis related changes and infertility lead to depression in diagnosed women. Depression changes lifestyle and induces physiological and immunological changes. A higher rate of depression and anxiety has been reported in women diagnosed with endometriosis, unleashing physiological, clinical and immune imbalances which further accelerate chronic endometriosis or vice versa. Thus, both endometriosis and depression are concomitantly part of a vicious cycle that enhance disease complications. A multidimensional treatment strategy is needed which can cater for both endometrial disease and depression and anxiety disorders.

## 1 Introduction

Endometriosis stands as one of the commonly encountered benign gynecological conditions in women, where endometrial glands and stroma exhibit extrauterine location, with a prevalence ranging from 6 to 10% among those of reproductive age (1, 2). Aberrant endometrial cells, characterized by genetic polymorphisms and proliferation rather than apoptosis, in response to local signals, lead to disease progression. Additionally, these cells when anomalously displaced into the peritoneal cavity, not only evade peritoneal destruction but also exploit the immediate environment to sustain proliferation in a clonal manner, while normal cells of the individual are systematically removed. Despite its non-malignant character, the inflammatory and erosive nature of the disease contributes to enduring alterations in a woman’s life, manifesting as persistent pelvic pain, dysmenorrhea, dyspareunia, and infertility (3). The disease can lead to additional symptoms such as painful bowel movements or urination, excessive bleeding, fatigue, diarrhea, constipation, bloating and nausea (4, 5). The challenge is exacerbated by the recurrent delay in diagnosis following the manifestation of symptoms and the restricted scope of available intervention strategies. Despite the potential existence of endometriotic lesions in asymptomatic women, a conclusive diagnosis of endometriosis is typically established when the presence of endometrial tissue or lesions is established beyond the confines of the uterus, frequently through surgical means (6). Endometriosis exhibits diverse classifications based on its anatomical location, including superficial peritoneal lesions which is the most common, ovarian endometrioma, deep sub-peritoneal infiltrating endometriosis and adenomyoma, which represents internal endometriosis within the myometrium (7). Endometriotic lesions have been identified in extra-pelvic locations, such as upper abdominal visceral organs, abdominal wall, diaphragm, and pleura, as well as within the nervous system (8). Patients may exhibit various forms concurrently.

The predominant classification method in use is an updated scoring system established by the American Society for Reproductive Medicine. This system is employed to ascertain the stage of endometriosis, denoted by Roman numerals I to IV, which represent the spectrum from ‘minimal’ to ‘severe’. It involves an assessment of type, location, appearance, depth of lesions, and an evaluation of overall extent of disease as well as presence of adhesions (9). However, grading using the ASRM criteria often demonstrates weak correlations of the abundance and location of lesions with the type of lesions, and symptoms of pain reported by patients, when compared to the disease stage. The occurrence of endometriosis in asymptomatic women, along with ambiguous reasons for its manifestation, contributes to varying perspectives on considering endometriosis as a ‘syndrome’ (10). Diagnosis is typically established only when a patient presents with both observable lesions and symptomatic manifestations.

An in-depth understanding of immune imbalance in endometriosis related depression and vice versa may include enhanced immune cell function, altered cytokine and chemokine levels and malfunctioning of regulatory proteins such as growth factors. Major immune cells such as macrophages, neutrophils, dendritic cells (DCs), natural killer cells (NK cells), T cells, and B cells exhibit great importance in the pathogenesis of endometriosis and depression. Increased levels of macrophages were observed in the peritoneal fluid of endometriotic patients (11). Neutrophil to lymphocyte ratio (NLR) was shown to be a clinically relevant indicator of endometriosis and associated outcomes. Increased NLR was also observed in a recent study showing higher numbers of neutrophils in endometriosis subjects (12, 13). Dendritic cells are important antigen presenting cells and in endometriotic patients, peritoneal DCs are found to increase. Furthermore, numbers of immature DCs are found to be greater as compared to mature DCs (14).

Endometriosis is associated with dysfunction in NK cell cytotoxicity and immunomodulation, by tolerating or inhibiting implantation, proliferation, and survival of endometrial cells, impairing their ability to eliminate these cells at ectopic sites (15). This review also sheds light on the role of the adaptive immune response in endometriosis, including helper T and B cells, whose roles remain incompletely understood. Several serum cytokines such as interleukins (IL) IL1β, IL-5, IL-6, IL-7 and IL-12 are involved, and their levels were found to be altered in endometriosis as compared to in healthy women (16). Cytokines IL-6, IL-8, IL-10, TNF-α also have important roles in the development of VEGF, which is involved in the pathogenesis of the disease (17–23).

Chronic stress or chronic depression events can modulate innate and adaptive immune responses with the involvement of enhanced inflammation and lowering the activity of immune protective cells (24). Inflammatory responses can be increased in stress (25). Furthermore, animal studies, showed that administration of proinflammatory cytokines (TNF or IL-1β) affect the central nervous system through decreased motor activity as well as increased social alienation, disturbed sleep patterns, altered appetite, reduced water intake and greater sensitivity to pain (26–28). Immune dysregulation and associated outcomes are the hallmarks of endometriosis. Immune dysregulation has also been shown to cause depression in susceptible individuals and hence may be the primary cause of depression in women with endometriosis.

Women diagnosed with endometriosis exhibit imbalanced immunological states often because of which major lifestyle changes are inevitable (29–31). Endometriosis patients may undergo mental health issues such as depression, physiological stress and anxiety (32). These women may bear day-to-day abdominal pain, painful bowel movements or urination, excessive bleeding, fatigue, diarrhea, constipation, bloating, nausea, fatigue and painful intercourse (4, 5), leading to a stressful life. In chronic cases infertility is very common (3, 33). This review aims to explore the potential links between depression, immunological factors and endometriosis.

### 1.1 Literature search for the review article

An electronic literature search was meticulously carried out by the authors S.S., M.W.A.K, S.R., K.M., Q.H., and W.A.K., as published by Centini et al. (34). The search team evaluated the existing literature on endometriosis, which included disease identification, symptoms, diagnosis, pathogenesis and immune dysregulations. The search was performed using the online medical MEDLINE database (accessed via PubMed). Terminologies included endometriosis, biomarkers, endometriotic symptoms and diagnosis, gynecological issues in endometriosis, pathogenesis in endometriosis, endometriotic depression. This review includes the most updated published articles as well as original articles which include randomized and non-randomized clinical trials, prospective observational studies, retrospective cohort studies, and case–control studies, review articles, and case reports. The selected articles were further checked for relevance with the aim and objective of the review. The bibliography of the selected articles was thoroughly checked for additional relevant articles. This procedure effectively helped in compiling more relevant, updated and high-quality peer-reviewed articles, providing a nuanced understanding of the specified topics “endometriosis, depression, and their associated immune imbalances.”

### 1.2 Etiology and incidence

Various physiological factors, including hormonal, metabolic, neurological, and immunological elements, play a role in the processes leading to the manifestation of symptoms. Epidemiological investigations reveal an increased susceptibility to various cancers (ovarian, breast and melanoma), rheumatoid arthritis, asthma and cardiovascular disease among women with endometriosis lesions (10). Endometriosis has familial incidence with heritability of up to 50% (35). It has been reported that having a first degree relative with a severe form of endometriosis raises the risk by up to seven times (36). A study focusing solely on relatives of individuals with endometriosis revealed that 16% of mothers and 22% of sisters of reproductive age had received a surgical diagnosis of endometriosis (36). Genome-wide association studies have found overrepresented single nucleotide polymorphisms (SNPs) in cases of severe disease. Gynecological disorders such as infertility, fibroids, and cancer were found to have overlaps with common SNPs associated with endometriosis, the etiology of which all involve steroid hormones (34, 37–40).

Additionally, five loci significantly associated with endometriosis risk were identified through a meta-analysis of 11 GWAS datasets, which genetically involved sex steroid hormone pathways (41). Irregularities in the role of extracellular matrix protein signaling such as fibronectin (42), laminin (43) and collagen (44) are implicated in abnormal cell migration and adhesion, contributing to fibrosis. Genomic studies have revealed associations between endometriosis and various biological pathways and cellular regulators. Notably, vezatin, a transmembrane adherens junctions’ protein, has been implicated (45), along with vascular endothelial growth factor receptor 2 (VEGFR-2) (46), the mitogen activated protein (MAP) kinase signaling cascade (47), IL1A (48), wingless related integration site (WNT) signaling (49), and steroid metabolism (50). Meta-analysis has highlighted common genetic signatures between migraine and depression in endometriosis, of which depression underscores an association with changes in gut mucosa (51). Additional determinants for endometriosis are low BMI, low birth weight, lower parity, Mullerian abnormalities, early menarche, short menstrual cycles or heavy and prolonged menstrual flow. Scientific evidence indicates variations in prevalence of endometriosis diagnosis across racial and ethnic groups. A systematic review revealed that Asian women exhibited an elevated risk, while Black women demonstrated a reduced risk compared to White women. However, it is plausible that these estimates may be influenced by biases linked to diagnosis and healthcare accessibility (52). The prevalence of endometriosis amongst Asian women of reproductive age is reported to range from 6.8% to as high as 16% (53) (see Table 1).

**Table 1:** Meta-analysis of genome wide association studies on endometriosis.

No.	Article	Reference	Gene/pathway
1	Gallagher et al., 2019	(37)	WNT4, CDC42, GREB1, ESR1, FSHB
2	Masuda et al., 2020	(38)	GREB 1, *LOC730100*, *PDE1C*, *TNRC6B*
3	Sapkota et al., 2017, 2015	(41, 50)	WNT4, GREB1, ETAA1, ILIA, KDR, ID4, 7p15.2, CDKN2B, VEZT, FN1, CCDC170, SYNE1, 7p12.3, FSHB

## 2 Clinical symptoms and diagnosis

Medical diagnosis of endometriosis is often difficult and delayed due to a lack of awareness and knowledge of the condition among healthcare professionals and limited understanding of its pathogenesis (35). Further, the complex nature of the disease as well as its manifestations, varying from asymptomatic to its evident phenotypes, add to a complicated diagnosis (3). Pelvic pain stands out as the primary indicator of endometriosis, manifesting in various forms such as dysmenorrhea, dyspareunia, or chronic pelvic pain (54). The intensity of pelvic pain is correlated with type of lesion classification and disease progression (55). Additional symptoms which are commonly found in individuals with the disease include abdominal discomfort, bloating, menometrorrhagia, lower back pain, and fatigue (3). Surgery remains the main method of obtaining a conclusive histopathological diagnosis, with Laparoscopy considered the gold standard diagnostic test. However, prevailing guidelines advocate for a non-surgical diagnostic approach reliant upon symptomatology, physical examination outcomes, and imaging findings. This strategy aims to mitigate delays in commencing treatment. In female patients undergoing surgical interventions, more than 50% will necessitate subsequent surgical interventions within a five-year timeframe (1). Numerous hormonal medical interventions are associated with adverse effects (56).

Research indicates that the greatest prevalence of endometriosis is observed between 25 and 29 years of age (57). However, there is often a significant diagnostic delay, with the average time from the onset of first symptoms to final diagnosis ranging from 4.4 years in the United States to 10.4 years in Germany (58, 59). The primary reasons for this delay may include intermittent use of contraceptives, misdiagnosis, and self-treatment of pain with over-the-counter painkillers. These findings align with the presented study’s results, which report a mean age of 26.9 years at the time of disease recognition and symptom onset ranging from 18.8 years for dysmenorrhea to 24.0 years for dyspareunia. This underscores the importance of early and accurate diagnosis to mitigate prolonged suffering and improve patient outcomes.

Central sensitization (CS) is a type of nociplastic pain characterized by a central nervous system response to peripheral nociceptive or neuropathic triggers, often seen in patients with chronic pains (60). Symptoms of CS include chronic pain, allodynia (pain from stimuli that do not usually provoke pain), hypersensitivity, hyperalgesia (increased sensitivity to painful stimuli), and mood changes (anxiety, panic attacks, and depression) (61–63). A Central Sensitization Inventory (CSI) score of 40 or higher has been effective in identifying CS in women with chronic pelvic pain, including those with endometriosis (62, 64). In a recent study, it has been showed that in endometriosis patients, CS can significantly worsen pain symptoms and is prevalent particularly among those with moderate to severe chronic pelvic pain, involvement of the posterolateral parametrium, high tone pelvic floor (HTF), and comorbid with central sensitivity syndromes like irritable bowel syndrome, anxiety, migraines or severe headaches (65). Therefore, recognizing and addressing CS is crucial for early and accurate diagnosis to mitigate prolonged suffering and improve endometriosis patient outcomes.

The need for reliable noninvasive biomarkers for the diagnosis, potential treatment response and disease prognosis persists as a significant unaddressed requirement. While certain types of endometriosis diagnosis can be expedited through imaging modalities, progress towards validating a dependable noninvasive blood test has been sluggish thus far (66). Other non-surgical diagnostic methods such as transvaginal ultrasonography and magnetic resonance imaging (MRI) have enabled identification of deep endometriosis types (67).

## 3 Hormones

**Figure 1:**
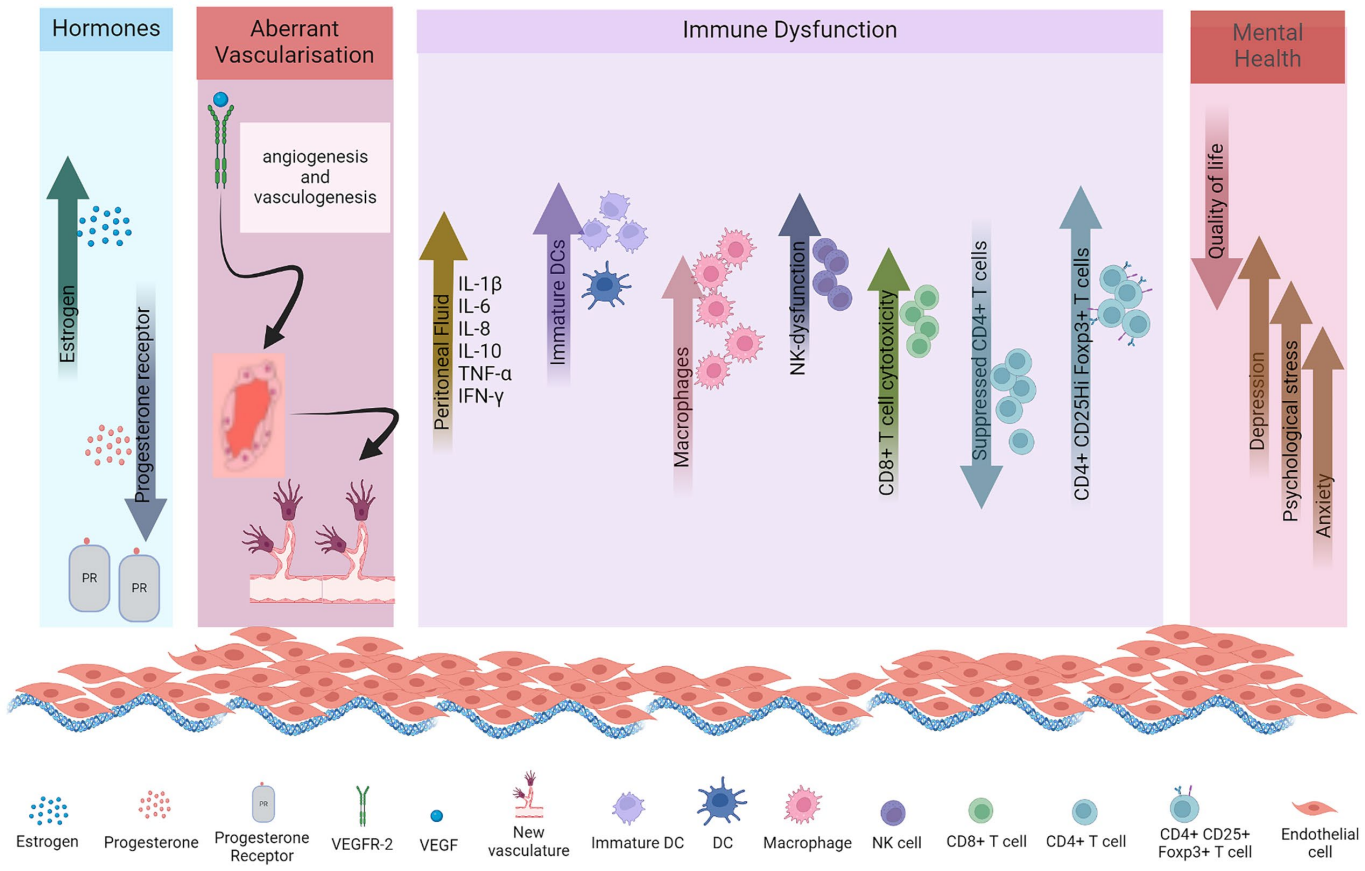
Pathogenesis of endometriosis, immune dysregulation, and mental health dysfunction.

Female sex hormones, estrogen and progesterone, play critical roles in the pathogenesis of endometriosis. Increased levels of estrogen with decreased progesterone receptor pathway signaling are implicated in disease pathogenesis (Figure 1).

### 3.1 Enhanced estrogen production

Elevated estrogen production consistently emerges as a dysregulated endocrine characteristic in eutopic endometrium and ectopic endometriotic lesions. The predominant estrogen, estradiol (E2), has a pivotal role in the post-menstrual endometrial regeneration (7). Both proliferation of endothelial cells and the re-establishment of microvasculature in this layer are orchestrated by E2, through interactions with its estrogen receptors (ERs), ERα and ERβ (68). Distinct intracellular localizations of the ERs lead to intricately coordinated and precisely regulated estrogen (E2) signaling pathways, which govern cellular proliferation, differentiation, and apoptosis. Endometrial E2 predominantly originates from the ovaries and, to a lesser extent, from adipocytes and the adrenal gland, transported to tissues through the circulatory system (69). Aromatase P450 (aromP450) is a rate limiting hormone in estrogen biosynthesis that catalyzes the conversion of androgens to estrogen, with subsequent transformation into E2 facilitated by 17β-hydroxysteroid dehydrogenase type 1 (*17*β*H*SDT1) (70). Prostaglandin E2 (PGE2) synthesis step is initiated by the rate-limiting cyclooxygenase-2 (COX-2) enzyme, acting on arachidonic acid, inducing dose-dependent aromP450 synthesis in endometriotic lesions (69). In healthy women’s endometrium, aromP450 activity is negligible (71). Cell-specific and menstrual cycle phase-dependent expression of receptors that bind to estrogens (ERα, ERβ and GPER1), androgens, progestins and glucocorticoids are observed in the healthy endometrium (72). However, both the endometrium and ectopic endometriotic lesions in women with endometriosis exhibit significantly elevated levels of aromP450, facilitating local E2 production. The capacity of the lesion to independently generate E2, coupled with the synthesis of the necessary enzymes, may enhance intraperitoneal endometriotic tissue implantation (56). It has been observed that the expression of ERβ is extraordinarily higher in stromal cells of women with endometriosis as compared to ERα. It is suggested that rather than just estrogen dependent, endometriosis should be considered steroid-dependent. Thus, the abnormal functioning of estrogen, its receptors, and estradiol synthesis-related enzymes is closely associated with endometriosis.

### 3.2 Progesterone resistance

Progesterone is the dominant hormone in the secretory phase of the menstrual cycle, where it counteracts effects of estrogen and prepares the uterus for supporting an embryo. It plays a decisive role in facilitating the differentiation of endometrial epithelial and stromal cells. Suppressed progesterone receptor (PR) expression, a characteristic feature of endometriosis, leads to resistance to progesterone and contributes to the development of severe endometriosis conditions (Figure 1). Endometriotic stromal cells demonstrate resistance to progesterone with reduced responsiveness to hormone (73). This diminished communication between stromal and epithelial cells leads to a subsequent elevation in the expression of ERβ within endometriotic lesions and stromal cells (1). PR-A and PR-B are the two functionally distinct receptor isoforms which interact with progesterone. In mice, the absence of PR-A results in abnormalities in the ovary and uterus, while the lack of PR-B has negligible impact on their function (74). Notably, the transcript for both receptor isoforms originate from the same gene, with PR-A having a shorter transcript than PR-B. This structure allows transrepression of PR-B and other nuclear receptors (75). Lesions in endometriosis exhibit a deficiency in PR-B expression, with minimal expression of the transrepressor PR-A, offering molecular substantiation for progesterone resistance. Subsequently, this leads to elevated local levels of estrogen (E2) as progesterone fails to stimulate 17β-hydroxysteroid dehydrogenase type 2 (17β-HSDT2) (69).

## 4 Aberrant vascularisation

The normal endometrium constitutes a steroid responsive tissue comprising richly vascularized epithelial and stromal cells as well as a diverse range of immune cells. Cells released from this tissue during menstruation encompass epithelial cells, stromal fibroblasts, vascular cells, and immune cells (neutrophils, monocytes, macrophages and uterine natural killer cells) (76). In retrograde menstruation, these cell types can potentially lead to lesions provided they maintain viability and evade the innate immune response and clearance within the intraperitoneal space. The three most implicated cells in peritoneal lesions are stem/progenitor cells, stromal fibroblasts, and immune cells, particularly stromal and immune cells, which play pivotal roles.

Endometriosis is postulated to originate due to endometrial fragment implantation within the peritoneal space. It potentially employs angiogenesis and vasculogenesis mechanisms to develop vascularization, essential for its sustenance (77, 78). The viability of endometriotic implants within the peritoneal cavity relies on establishing a blood supply to deliver oxygen and nutrients to the developing lesions. Concurrent with endometrial growth, the endometrial vasculature undergoes cyclical proliferation and regeneration orchestrated by ovarian steroids, particularly E2. Vascular endothelial growth factor (VEGF) serves a pivotal function in initiating angiogenesis in endometriosis, particularly in ectopic lesions (69, 79). As a vasoactive agent, it participates in numerous physiological functions, such reestablishment of a vascular network and subsequent healing of the uterus, by modulating proliferation and migration of endothelial cells. Heightened expression of VEGF mRNA in the superficial endometrial layer was reported during both the two phases of the uterine cycle, i.e., proliferative and secretory, suggesting ongoing angiogenesis (80). Furthermore, it was also demonstrated that estradiol was responsible for stimulating expression of VEGF in endometrial cells. Administration of E2 resulted in elevated levels of VEGF mRNA expression compared to endometrial cells not exposed to E2 stimulation. Given the intrinsic angiogenic capacity of healthy endometrium regulated by estradiol, it becomes apparent that dysregulated VEGF expression and E2 levels promote neovascularization in lesions, facilitating their establishment in ectopic sites. Studies indicate that peritoneal fluid (PF) from subjects with advanced endometriosis harbors elevated VEGF concentrations versus those with mild disease or healthy individuals (81). Various immune cells participate in angiogenesis by generating and subsequently increasing levels of proinflammatory and angiogenic cytokines, as well as cellular adhesion factors within the PF, surrounding endometriotic lesions. Secretion of VEGF by neutrophils and macrophages within intraperitoneal lesions facilitates angiogenesis (82). Disruptions in peritoneal homeostasis, coupled with the induction of proinflammatory and proangiogenic cytokine production in endometriosis, collectively contribute to modified innervation and the modulation of pain pathways in affected individuals (54). DCs have also been linked to angiogenesis (83). This was evidenced by a study revealing heightened perivascular localization of VEGFR-2 secreting immature dendritic cells within such lesions. These DCs exhibited the ability to stimulate endothelial cell migration *in vitro*. Intraperitoneal DCs in the peritoneal cavity led to the development of endometriotic lesions in the murine model (84). An investigation employing a transgenic murine model featuring diphtheria toxin mediated conditional depletion of DCs, scientists observed that endometriotic lesions in DC-depleted mice exhibited notable increased size versus control counterparts, along with reduced CD69 expression, indicative of antigen stimulated T and natural killer cell activation. These results underscore the direct involvement of DCs in regulating the angiogenic process and modulating immune activation subsets during the development of lesions (85). Endometrial cells exhibit enhanced resistance to cell mediated immunity, alongside enhanced proliferation and heightened aromatase expression, culminating in elevated estrogen levels (69, 70, 86).

Comparative studies investigating stromal fibroblast phenotypes in women with endometriosis have revealed behavioral disparities, notably epigenetic alterations leading to aberrant responses to estrogen (87). It is plausible that cell plasticity evolved to expedite endometrial repair post-menstruation, leading to multicellular lesion formation in extrauterine locations. Mechanistic similarities between menstrual regulation and lesion formation encompass transient hypoxia (88), iron release, and platelet activation (89, 90).

## 5 Immune dysfunction

Endometrial lesions adhere to the peritoneum or are closely associated with the ovaries, exposing them to an altered peritoneal environment comprising immune cells, cytokines, and regulatory proteins such as growth factors, with a high potential for anomalous behavior of these entities. Endometriosis animal model studies are suggestive of the fact that immune cells within lesions consist of a combination of cells from endometrial shedding as well as cells from peritoneal microenvironment (91). Fragments of endometrial tissue elicit intraperitoneal inflammation, which results in activation and recruitment of neutrophils and macrophages to the area. Hence, women with the disease often exhibit elevated concentrations of activated macrophages secreting proinflammatory and chemotactic cytokines in the peritoneal fluid (92). Given that various estrogen receptors are expressed on both macrophages and nerve fibers, estrogen is postulated to modulate macrophage and nerve fibers behavior. Thus, estrogen regulation encompasses macrophage recruitment, atypical neurogenesis atypical inflammation observed in endometriosis (93).

### 5.1 Cytokines

Several studies were conducted for the involvement of cytokines in the pathogenesis of endometriosis (Figure 1) (16–23). Multan et al., found that serum cytokines IL1β, IL-5, IL-6, IL-7 and IL-12 levels were elevated in serum samples of endometriosis patients compared to normal women (16). Cytokines (IL-6, IL-8, IL-10, TNF-α) and chemokines (CCL-2) as well as growth factor VEGF increased in peritoneal fluid of patients (18–23).

Nerve fibers demonstrate an exceptional capacity to recruit macrophages to the injury site. Numerous mediators identified in this process including leukemia inhibitory factor, IL1α, IL1β (94) and pancreatitis-associated protein 3 (PAP3) (95). Estrogen has also been shown to promote colony-stimulating factor 1 and C-C motif ligand 2 (CCL2) secretions from PNS, thereby amplifying macrophage movement towards lesions (96). Additionally, macrophages contribute to the proliferation of peritoneal implants and act as significant sources of angiogenic factors like TNF-α and IL-8. They also contribute to hypoxia-induced angiogenesis (92).

Endometriosis, like cancer, can be categorized as a metabolic disorder. Under the influence of transforming TGF-β1, tumor cells adopt aerobic glycolytic phenotype, leading to enhanced lactate secretion and accumulation (97). Elevated levels of TGF-β1 and lactate are observed in endometriotic PF. Concurrently, there is a shift from typical mitochondrial phosphorylation to glycolysis in the mesothelial cells lining the peritoneum to support cell survival in a tumor like microenvironment (98). Like in tumorigenesis, endometrial cells also exhibit the Warburg effect, where cells adjacent to tumors exhibit a programmed utilization of aerobic glycolysis induced by TGF-β1, leading to lactate production. This lactate serves as a nutrient source for neighboring tumor cells, thereby establishing a cohesive metabolic microenvironment conducive to tumor progression (99). Lactate induces lactylation or the covalent modification of lysine residues on histones and other proteins. Research findings indicate that elevated levels of lactate and lactate dehydrogenase-A, contribute to enhanced lactylation of histone H3 lysine 18 in ectopic endometrial tissues and ectopic endometrial stromal cells, compared to normal cells (100). Furthermore, lactate promotes cell proliferation, migration, and invasion in endometriosis progression, further linked to immune suppression and possible transformation to a malignant form.

### 5.2 Macrophages

Macrophages represent the predominant immune cell population in the peritoneum. Alterations in macrophage phenotype, or polarization, are linked to significant metabolic shifts. The peritoneal fluid of patients with endometriosis exhibits increased levels of macrophages (11), as shown in Figure 1. These macrophages do not effectively clear endometrial tissue; instead, they significantly contribute to high levels of cytokines (95). Proinflammatory macrophages primarily rely on glycolysis, whereas anti-inflammatory M2 macrophages exhibit a greater dependence on oxidative phosphorylation (101). Moreover, macrophages produce angiogenic mediators, such as TNF-α and IL-8, thereby promoting the growth of lesions (102). While macrophages appear to play a role in the growth and development of endometriotic tissue, depletion of macrophages does not prevent the implantation of endometrial cells in the peritoneum.

### 5.3 Neutrophils

Neutrophils are postulated to essay a pivotal role in endometriosis pathogenesis. Neutrophils significantly contribute to the resolution of inflammatory responses. A study found that when neutrophils from healthy women were exposed to endometrial plasma or PF, reduced neutrophil apoptosis was observed versus controls, elucidating the presence of antiapoptotic factors in the plasma and PF (12). Interleukin-8 stood out in the study due to its proinflammatory nature and its involvement in neutrophil chemotaxis during inflammation (12).

### 5.4 Dendritic cells

Dendritic cells are antigen-presenting cells which initiate and modulate adaptive immune responses. DCs additionally serve a crucial function in the prevention of autoimmunity by functioning as mobile sentinels. They transport self-antigens to naïve T cells residing in lymphoid organs, thereby facilitating the induction of self-tolerance (103). In healthy women, immature dendritic cells are absent from the peritoneal membrane. In endometriosis they are present within endometriotic lesions and adjacent to peritoneum. Additionally, the numbers of mature DCs are significantly reduced in the endometrium throughout the menstrual cycle in women with endometriosis compared to those with healthy endometrium (Figure 1). Endometriotic conditions may impede the maturation of immature DCs and prompt their transition into a macrophage phenotype. Moreover, the progression and vascularization of lesions necessitate the presence of endogenous DCs, which infiltrate these lesions and augment endothelial cell migration through the secretion of proangiogenic factors (104). In murine models, the cell density of peritoneal dendritic cells increased promptly following the injection of endometrial tissues, peaking at 14 days. The proportion of mature DCs within peritoneal DCs initially decreased post-injection, then gradually rose over time, although remaining lower than the control group at 42 days. Conversely, the proportion of immature DCs exhibited contrasting changes (14). The administration of lipopolysaccharide resulted in a significant increase in mature DCs proportion, consequently leading to reduced volume and weight of endometriosis lesions. While DC maturation suppresses the angiogenic response, immature DCs actively promote angiogenesis and lesion growth, thus undergoing a shift in their immunological function from antigen presentation to supporting angiogenesis and the progression of the disease.

### 5.5 Natural killer

NK cells are cytotoxic effector lymphocytes of the innate immune response characterized by their capacity to induce lysis of target cells independent of prior antigen exposure. Endometriosis is associated with a dysfunction in NK cell cytotoxicity and immunomodulation, by tolerating or inhibiting implantation, proliferation, and survival of endometrial cells, impairing their ability to eliminate these cells at ectopic sites (15). A study identified soluble immunosuppressive factors present in the media of both normal endometrial cells and endometriotic stromal cells. Healthy endometrium possesses immunosuppressive capabilities against NK cell cytotoxicity, potentially facilitating embryo implantation (Figure 1). However, in endometriosis, the immunosuppression is more pronounced, potentially allowing retrogradely displaced endometrial tissue to develop into lesions within the peritoneal environment (105). Functional defects and dysregulation of NK cell cytotoxicity are attributed to various cytokines and inhibitory factors present in both serum and PF. The reduction in NK cytotoxicity appears to result from functional defects. The dysregulated cytotoxicity of peritoneal NK cells in endometriosis can be attributed to various cytokines (IL-6, IL-8, IL-1β, IFN-*γ*, and TNF-*α*) and inhibitory factors present in both serum and peritoneal fluid. Also, for such patients, there is a notable reduction in the populations of mature NK cells (CD32CD56+), while immature NK cells are elevated in the PF, leading to apoptosis (106). The observed abnormalities in NK cells among women with endometriosis may indeed be outcomes resulting from the local regulation of microenvironment due to the pathology itself.

Treatment modalities such as inhibition of receptor-ligand interactions involving KIR2DL1, NKG2A, LILRB1/2, and PD-1/PD-L1, TGF-β; stimulation of NK cells via IL-2; and mycobacterial therapy utilizing Bacillus Calmette-Guérin (BCG) (82, 107–109). Moreover, ongoing research is exploring the potential of adoptive NK cell therapy for managing endometriosis. Endometriosis holds promise as a candidate for immunotherapy aimed at blocking negative regulatory checkpoints of NK cells, such as inhibitory NK cell receptors. Attenuating the cellular cytotoxicity of NK cells could potentially mitigate the progression of pelvic pain in individuals affected by the disease. The principal inhibitory receptors on NK cells, which are potential checkpoints for eradication of ectopic endometrial tissue, are leukocyte immunoglobulin-like receptors (LILRs).

### 5.6 T and B cells

Adaptive immune response entails helper T and B cells, in endometriosis, which remains incompletely understood. A study showed that a higher number of CD8 T cells are present in endometriotic lesions compared to eutopic endometrium (110). However, in blood circulation the CD8 T cell populations show no difference between patients and healthy women. It has been noted that CD8 T cell cytotoxicity is enhanced in menstrual effluent of patients, specifically CD8 T effector memory cells are enriched in eutopic endometrium of patients (Figure 1) (110).

Suppressed CD4 T cells have been reported in endometriosis due to the systemic and local alterations in immune responses (Figure 1). These impaired CD4 T cells potentially contribute to the pathogenesis of endometriosis disease through cytokines, which are important for implantation and proliferation of ectopic endometrial cells, inflammation and angiogenesis (111). In women with this condition, there appears to be a bias towards Th2 cell polarization, as evidenced by robust intracellular IL-4 expression and the absence of IL-2 in ectopic lesion derived lymphocytes (82). The equilibrium of CD4 cells in endometriosis remains contentious, with studies indicating reduced activation of both Th1 and Th2 cells in the peritoneal fluid of affected individuals (110).

Regulatory T (Treg) cells constitute a distinct subset within the T cell population, balancing immunological self-tolerance and homeostasis, thus modulating the immune system’s response to prevent excessive reactions against the host (97). Nevertheless, the precise involvement and significance of Treg cells in the context of endometriosis remain inadequately elucidated. The Forkhead box 3 protein (Foxp3), identified as a pivotal transcriptional factor, serves as a master regulator gene governing the differentiation of CD4+ Treg cells (112). Berbic et al. (113), demonstrated heightened expression levels of Foxp3 within both eutopic and ectopic endometrial tissues during the secretory phase of the menstrual cycle in patients afflicted with endometriosis. Furthermore, elevated Foxp3 expression at the messenger RNA level within ovarian endometrioma tissue (114), along with a relatively higher ratio of CD4 + Foxp3+ cells within the CD4+ cell population (115).

Additionally, recent studies have shown a significant increase in the proportion of CD4 + CD25hiFoxp3+ cells within the PF, but not in peripheral blood, of endometriosis patients, as opposed to those without the disease (Figure 1) (116, 117). These collective findings proved the abundance of Treg cells within localized endometrial lesions, implicating their potential involvement in the pathophysiology of endometriosis.

Additionally, heightened activation of B cells has been observed in both eutopic endometrium and lesions compared to healthy endometrium. Notably, the presence of anti-endometrial antibodies in the serum of endometriosis subjects has led to its occasional classification as an autoimmune disease (118).

### 5.7 Stem cells

Traditional hypotheses concerning the development of endometriotic lesions have lacked detailed mechanistic explanations for their proliferation and survival until recent studies revealed the involvement of mesenchymal stem cells (MSCs) and myeloid-derived suppressor cells (MDSCs) within a complex network of immune-endocrine signaling. MDSCs typically have strong immunosuppressive and angiogenic characteristics and are found in low numbers in healthy tissue. However, their accumulation is linked to interactions with inflammatory cytokines and has been implicated in several inflammatory diseases. Increased levels of these pro-inflammatory cytokines within the PF of individuals with endometriosis-associated pain may influence the differentiation of monocytes into MDSCs (119).

## 6 Immunological pathogenesis of endometriosis

Estrogen dominance fosters immune dysregulation, whereby many features observed in endometriosis mirror immune processes observed in various cancers, including heightened somatic mutations in endometrial epithelial cells. This elevated mutational burden contributes to the development of endometriosis-specific neoantigens, potentially altering the immune microenvironment of the lesions. Additionally, endometriosis often coexists with several chronic inflammatory conditions, characterized by shared dysregulation of the IL-23/IL-17 pathway, as evidenced in inflammatory bowel disease, psoriasis, and rheumatoid arthritis (120).

The crosstalk between immune cells, nerves, and central pain pathways plays a significant role in the pathophysiology of endometriosis. Endometrium is unique among mucosal tissues in the body in that it typically lacks innervation under normal physiological conditions. Nerve fibers are rare within the functional layer of the endometrium in women without any pathology (121). Sensory nerves surrounding endometriotic lesions drive the chronic pain associated with the condition and contribute to a pro-growth phenotype (122). Substantial alterations in nerve activity occur both within endometriotic lesions and the nervous system. Studies indicate that women experiencing pain symptoms associated with endometriosis exhibit notably higher nerve fiber density within the endometrium, myometrium and lesions as compared to those without the condition (123). Nerve fibers within endometriotic lesions consist of a combination of sensory, sympathetic, and parasympathetic fibers, collectively contributing to pain and inflammatory processes (124). The pain associated with endometriosis implies neuronal mechanisms that culminate in CS.

The interplay between macrophages and nerve fibers fosters inflammation and pain manifestations in endometriosis. Given their abundance within endometriotic lesions, macrophages stimulate sensory innervation and sensitization, thereby contributing to lesion proliferation and the prevalent pain experienced in endometriosis (19, 23). Moreover, immune cells release pro-nociceptive and pro-inflammatory mediators that can sensitize nerve fibers, leading to neurogenic inflammation (125). This communication between immune cells and nerves presents promising avenues for therapeutic interventions in endometriosis.

Prostaglandins, particularly prostaglandin E2 (PGE2), also play a significant role in the pathophysiology of endometriosis, contributing to pain and inflammation. Women with endometriosis produce an excess of PGE2, which is responsible for uterine contractions, pain, and inflammation (126). PGE2 is upregulated in the peritoneal cavity in endometriosis and is produced by macrophages and ectopic endometrial cells (127). It is involved in the development and continued growth of endometriosis, as it increases estrogen synthesis, inhibits apoptosis, promotes cell proliferation, affects leukocyte populations, and promotes angiogenesis (127). The presence of endometriosis lesions can trigger inflammation, which further promotes PGE2 activity (128). The release of PGE2 is associated with the development of symptoms and the progression of endometriosis, making it a potential target for therapeutic interventions. These changes in nerve activity contribute to the complex and debilitating pain experienced by individuals with endometriosis. However, the use of painkillers or non-steroidal anti-inflammatory drugs (NSAIDs) alone is not always ideal for managing endometriosis pain, as they may have limited efficacy and potential adverse effects (1). Therefore, understanding the role of PGE2 in endometriosis is important for developing targeted treatment strategies to address the associated pain and inflammation.

## 7 Clinical consequences of depression in endometriosis

**Figure 2:**
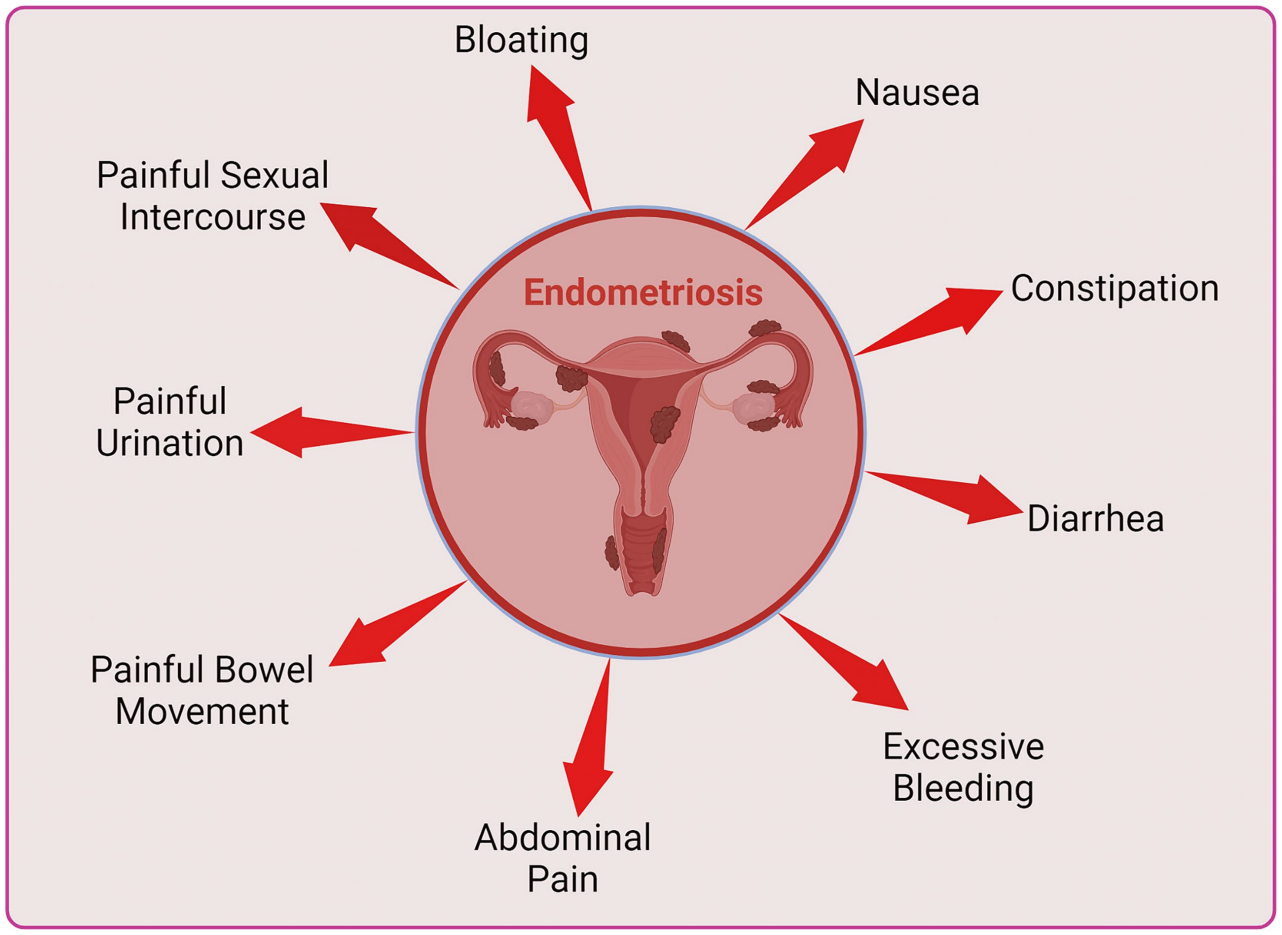
Various health issues in individuals with chronic endometriosis.

Endometriosis changes the lifestyle of women and may lead to mental health issues such as depression, physiological stress and anxiety as depicted in Figure 1. Endometriosis is linked to psychological disorders in several ways. The disease in chronic stage can cause life impacting abdominal pain during periods, painful bowel movements or urination, chronic pelvic pain, excessive bleeding, fatigue, diarrhea, constipation, bloating, nausea, fatigue and painful intercourse (Figure 2), leading to a compromised quality of life and in several cases infertility. These co-occurring conditions may cause stress, anxiety and psychological disorders (4, 5).

A study conducted by Pope et al. (129) highlighted the correlation between endometriosis and a diverse array of psychiatric symptoms, notably depression, anxiety, psychosocial stress, and diminished quality of life. Recent literature further substantiates the prevalence of depression and anxiety as the predominant psychiatric comorbidities in individuals with endometriosis (129–136). In an investigation by Low et al. (137), for the potential role of a distinct psychological profile associated with endometriosis, the author included 81 women participants in the study who were experiencing pelvic pain. Of these, 40 were diagnosed with endometriosis disease and 41 presenting with alternative gynecological issues. All the subjects underwent evaluation through six standardized psychometric assessments, including the Eysenck Personality Questionnaire (EPQ), Beck Depression Inventory (BDI), General Health Questionnaire, State–Trait Anxiety Inventory (STAI), The Golombok Rust Inventory of Marital State, and The Short-Form McGill Pain Questionnaire. In assessments using these criteria, the endometriosis patients exhibited increased level of psychoticism, introversion and anxiety scores than women with other gynecological issues (138).

In a recent study conducted by Warzecha et al. (139), 15.1% of women with endometriosis were diagnosed with depression which aligns with findings by Fried et al., who reported a 14.5% incidence of depressive symptoms. In another study the incidence of symptoms of anxiety were estimated to be 29% among Austrian women with endometriosis (130). A meta-analysis by Gambadauro et al. (140), encompassing 24 studies and 99,614 women, confirmed higher levels of depression in such subjects. Researchers indicate that women with endometriosis accompanied by pelvic pain, the rate of depressive symptoms is significantly higher than in cases of endometriosis without pain. This evidence suggests that endometriosis associated complications such as pain may be a more critical factor in the development of depressive symptoms than the presence of endometriosis alone (140). Furthermore, Warzecha et al. (140), revealed that the mean age at the onset of depressive symptoms among women with endometriosis was 22.2 years, which is closely aligned with the age range for the onset of endometriosis symptoms between 18.8 and 24 years. Additionally, the study found that certain types of pain, specifically chronic pelvic pain and painful defecation, significantly increased the incidence of depressive symptoms (140). These findings underscore the profound impact that specific pain manifestations can have on the mental health of women suffering from endometriosis. In these studies, clinicians caring for women with chronic pelvic pain, particularly when coexisting with endometriosis, should be cognizant of the elevated risk of depressive disorders in this population. Understanding the strong correlation between chronic pain and mental health is essential for providing holistic care. Early recognition and intervention for depressive symptoms in these patients can significantly improve their overall quality of life and treatment outcomes.

Another study based on meta-analysis included 18 relevant quantitative studies (129). Out of the 18 studies, 17 included clinical patients’ samples. Fourteen out of eighteen studies indicated that endometriosis or chronic pelvic pain significantly impaired at least some aspects of psychological functioning, mental health, elevated risk for depression, hypomanic, or anxiety symptoms among affected women.

From the 18 studies, 4 studies (137, 141–143) used clinical diagnostic criteria to assess psychiatric diagnosis. Out of these 4 studies, 3 were used as comparator group (137, 141, 142). From the clinical samples of women (age from late teens to mid-40s), 37% of participants showed endometriosis and 50% exhibited pelvic pain with a reported family history of mood disorders. From the 3 comparator group studies, 2 showed higher risk of psychiatric disorders in women with endometriosis (137, 141). Data from these three studies exhibited that 44 (56%) of the 79 women with endometriosis met the criteria for at least one psychiatric disorder.

Another study was conducted on a Brazilian population including 103 women with an age range of 15 to 49 years (average age 33.4 years) (144). Out of 103 patients, 53 (51.5%) were diagnosed with endometriosis and 50 (48.5%) without endometriosis (control). Subjects were evaluated using a questionnaire (Beck Depression Inventory) providing different levels of depression (mild, moderate, moderate to severe, and severe). Based on the questionnaire, symptoms for depression were observed in 35 (66%) women with endometriosis. Out of these, 20 (37.7%) women showed mild depression, 4 women (7.5%) exhibited mild to moderate, 6 women (11.3%) were found to have moderate to severe depression, and 5 (9.4%) women had severe depression. However, according to the Fisher’s exact test, there was no relationship between endometriosis and depressive symptoms (*p* = 0.423) (144).

Traumatic stress is very likely in endometriosis diagnosed women compared with the women without endometriosis (145, 146). Harris et al. concluded in a study that children who experienced physical or sexual abuse were likely to develop endometriosis in later stages of life (147). Post-traumatic stress disorder and childhood trauma can impact individuals and may contribute to the development of depression at an early stage (148). Furthermore, a study conducted by Reis et al., showed that depression or stress in the early stages of life may be considered an important factor for the development of endometriosis (149). The persistence of such conditions over a long period of time may lead to hormonal imbalance, neuroendocrine dysfunction, chronic inflammation which are leading factors in the development of depression and endometriosis (146, 150).

## 8 Immunological aspects of depression in endometriosis

Depression is very much associated with the secretion or formation of proinflammatory molecules such as IL-6, IFN-γ, TNF-α, and IL1β (151–153). Additionally, depression also enhances oxidative stress and increases oxidative molecules such as protein bound carbonyl content and methylglyoxal (151, 152, 154). A study revealed that methylglyoxal, which is a well-known reactive metabolite, plays a vital role in various central nervous system associated cognitive functions and can be linked to stress, depression, anxiety, and neurodegenerative diseases (154, 155). Numerous studies conducted in this area have proven that endometriosis is strongly linked with the increased risk of psychological depression, anxiety, and eating disorders (156).

Studies indicated that endometriosis patients have an increased incidence of autoimmune diseases and cancer (157–159). Women diagnosed with endometriosis are more prone to several autoimmune diseases such as multiple sclerosis, rheumatoid arthritis, inflammatory bowel disease, systemic lupus erythematosus, and Sjogren’s Syndrome (159, 160). Higher estrogen levels in women during endometriosis lead to the modification of macromolecules like insulin, serum albumin etc. (161–163). These modifications not only compromise the functioning of these macromolecules but also lead to the formation of neo-antigens on these molecules that activate a cascade of the reactions causing production of autoantibodies (161–163). Higher levels of autoantibodies were detected in patients with depression (161–163). These elevated levels of autoantibodies, together with several pathological complications in endometriosis as discussed above, further aggravate the disease to extremely severe levels.

T cell involvement in depression has not been investigated in detail. Some studies conducted in this area revealed that T cell responses decrease in depression (164–166). T cell responses were found to decrease against antigens encountered in the skin of depressed individuals (164, 167). In a meta-analysis conducted by Zorrilla et al. (166), depression was associated with a decreased percentage of T cells. CD4+ T cells in depressed individuals exhibited increased expression of Fas (CD95) which is known as death receptor as it triggers apoptosis when it interacts with its ligand (168, 169).

T cell function can be inhibited by glucocorticoid pathways in major depression. Increased levels of glucocorticoids in circulatory blood are hallmark of depression (170). Glucocorticoids mediate cell migration and induce apoptosis of immune cells including T cells (166, 171). Endometriosis is characterized by elevated expression of the *HSD11B1* gene, which converts inactive cortisone to cortisol, a biologically potent glucocorticoid in peripheral tissues. Receptor for glucocorticoid expression increases up to 3.5-fold in endometriosis. The interaction of higher levels of glucocorticoids with increased level of receptors in endometriosis may increase the proinflammatory environment surrounding the endometriotic lesion and enhance the activity that supports endometriotic cell survival (172). Higher levels of glucocorticoids also induce infertility in women. Infertility treatment, which are often long and painful processes, as well as the condition itself induce depression and compromises in quality of life.

**Figure 3:**
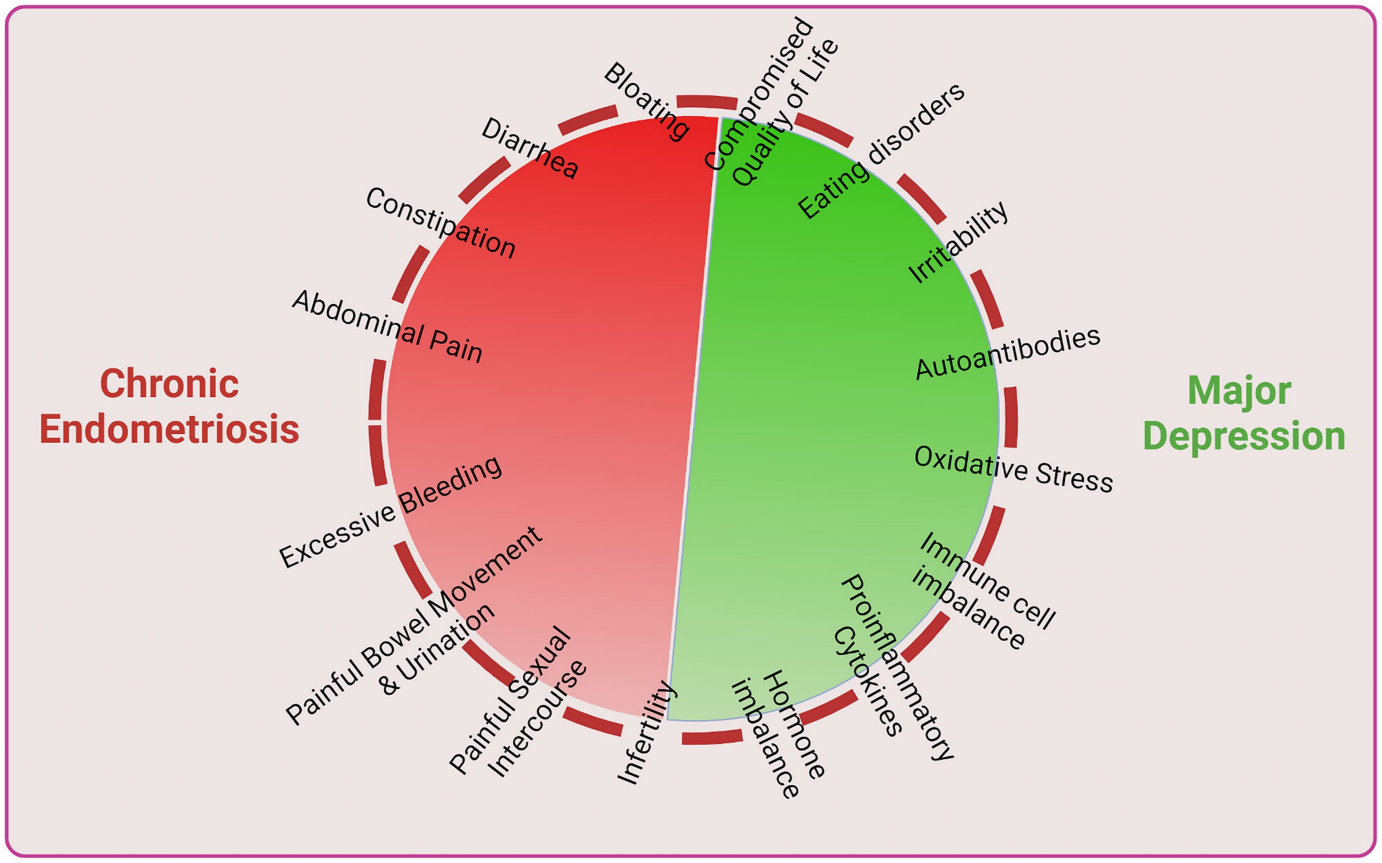
Interlink between chronic endometriosis and major depression.

Finally, it has been suggested that chronic endometriosis arises due to various dysfunctions imbalances and may lead to infertility which may cause women to develop depression and subsequently unleash further physiological, clinical and immune imbalances which further accelerate chronic endometriosis or vice versa (Figure 3). Thus, both endometriosis and depression concomitantly develop a vicious cycle which enhance and exacerbate disease complications.

## 9 Links between depression and immunological factors for potential malignant transformation of endometriosis

Although endometriosis is classified as a benign disease, it has the potential to transform into malignancy, which occurs in about 1% of endometriosis patients (173, 174). This malignant transformation most frequently affects the ovaries, with ovarian endometrioid carcinoma and ovarian clear cell carcinoma being the most common types. These two malignancies account for 76% of all endometriosis-related ovarian cancers (174, 175).

Recently, several carcinogenic pathways have been identified for endometriosis-related malignant transformation. Uncontrolled cell division, tissue infiltration, neoangiogenesis, and apoptosis evasion may result from oncogene demethylation and tumor suppressor gene hypermethylation (173, 174). Key events include hypermethylation of the hMLH1 gene promoter, reducing DNA mismatch repair gene expression, and hypomethylation of LINE-1. Tumor suppressor genes *RUNX3* and *RASSF2* are inactivated by promoter hypermethylation (173). In endometrioid cancer, *KRAS* oncogene activation and *PTEN* tumor suppressor gene inactivation is significant (175, 176). Loss of *PTEN* activity, an early event in malignant transformation, is linked to *PTEN* gene mutations (177). Additionally, somatic mutations in cancer driver genes *ARID1A*, *PIK3CA*, *KRAS*, and *PPP2R1A* are found in deep infiltrating endometriosis (178).

A recent meta-analysis study conducted by Centini et al. (34), focusses on atypical endometriosis, which is present in 12–35% of ovarian endometriosis cases and 60–80% of endometriosis associated ovarian cancers. The *SWItch/Sucrose Non-Fermentable* (*SWI/SNF*) complex and *ARID1A* gene alterations offer valuable insights into the pathogenesis of endometriosis and endometriosis-associated ovarian cancer. Also, the use of potential therapeutics based on inhibitors and suggested the use of PARP inhibitors in treating ovarian cancer which may potentially improve outcomes for these conditions.

Retrograde menstruation, where menstrual blood containing erythrocytes, macrophages, and endometrial tissue travels through the fallopian tubes to the peritoneal cavity, is crucial for understanding endometriosis pathogenesis (179, 180). Periodic hemorrhage from ectopic endometriotic lesions causes iron overload, with erythrocyte-derived iron being a well-known inducer of oxidative stress (180). This altered iron metabolism can contribute to endometriosis development and progression (181). At moderate levels, iron-induced reactive oxygen species (ROS) stimulate ectopic endometrial cell proliferation, angiogenesis, and adhesion. Animal models show that iron treatment increases the number and size of endometriotic lesions compared to controls, suggesting that imbalances in iron homeostasis regulate endometriotic cell proliferation (182). These finding suggest that alterations in iron hemostasis may promote endometriotic cell proliferation. Iron overload intensifies intracellular oxidative stress through the Fenton reaction (Fe^2+^ + H₂O₂ → Fe^3+^ + OH^−^ + OH), leading to DNA, lipid, and protein damage, and resulting in cytotoxic effects on cells (183). This reaction generates reactive hydroxyl radicals that contribute to cellular injury and dysfunction. Furthermore, excess iron can decrease transferrin concentration in follicular fluid due to increased transferrin saturation. This iron overload and transferrin insufficiency lead to elevated ROS levels, compromising mitotic spindle integrity and promoting chromosome instability (184, 185). Consequently, this may affect the number and maturation of oocytes retrieved from women with endometriosis (184, 185). High content of iron in ovarian endometriomas exert negative effect on granulosa cells via increased level of ROS cause decrease in the number and quality of oocytes leading to impaired fertility (186–188). The increased levels of free radical generation in physiological stress concomitant with impaired fertility in endometriosis may be due to an imbalance in ROS homeostasis.

Furthermore, there is a persistent production of antioxidants, where endometriotic cells adapt to oxidative stress with the support of macrophages. This adaptation enhances antioxidative defenses and influences redox signaling, energy metabolism, and the tumor immune microenvironment, potentially leading to malignant transformation. Moreover, specific molecular alterations, including mutations in *ARIDA1/BAF250a, PIK3CA, CTNNB1*, and *PTEN*, as well as microsatellite instability and loss of heterozygosity, have been reported (189–193).

## 10 Conclusion

Endometriosis is a chronic, estrogen-dependent, proinflammatory disease that can cause various dysfunctions. Hormonal imbalance, inflammation, immune dysregulation, angiogenesis, neurogenic inflammation, epigenetic alterations, and tissue remodeling are common in the pathogenesis of endometriosis. Higher numbers of women diagnosed with endometriosis showed increased levels of depression which can potentially further aggravate the disease. According to published literature strong synergisms were observed in endometriosis patients with depression or vice versa. This review article focuses on the immunological aspects of depression in endometriosis patients by looking at the links between depression and immunological factors responsible for potential malignant transformation of endometriosis. There is a huge gap in the awareness of endometriosis and proper counselling and treatment, especially in underdeveloped and developing countries due to continued reluctance of open discussion of female gynecological issues. Clinicians, academicians, and scientists should reach out to these communities and provide vital information promoting regular screening, early detection of the disease and counselling to prevent further complications. Importantly, increased funding is critical for investigation and identification of factors against which multifactorial drug development is critical to alleviate the pain and suffering of women diagnosed with endometriosis and depression.

## Author contributions

SS: Conceptualization, Data curation, Supervision, Writing – original draft, Writing – review & editing. MK: Data curation, Writing – original draft, Writing – review & editing. SR: Data curation, Writing – original draft. KA-M: Data curation, Writing – review & editing. QH: Data curation, Writing – review & editing. WK: Data curation, Writing – review & editing.
